# The Effects of Thoracic Flexibility Exercise on Pain, Range of Motion, Spinal Alignment, Proprioception, and Neck Disability Index in Neck Pain Patients with Forward Head Posture: A Randomized Controlled Trial

**DOI:** 10.3390/medicina62091666

**Published:** 2026-08-31

**Authors:** Changhoon Kim, Donghwan Park

**Affiliations:** Department of Physical Therapy, Graduate School, College of Health Science, Kyungnam University, Changwon-si 51767, Republic of Korea

**Keywords:** neck pain, cervical vertebrae, thoracic vertebrae, exercise therapy

## Abstract

*Background and Objectives:* Thoracic dysfunction may contribute to neck pain, restricted mobility, and altered spinal alignment in individuals with forward head posture (FHP). This study compared thoracic flexibility exercise (TFE) with cervical stabilization exercise (CSE) in terms of their effects on pain, range of motion (ROM), spinal alignment, proprioception, and neck-related disability in patients with chronic neck pain and FHP. *Materials and Methods:* Twenty-four participants were randomly assigned to either the TFE group (*n* = 12) or the CSE group (*n* = 12). Both groups performed their assigned intervention once per day, three days per week, for four weeks. The outcome measures included pain intensity, cervical and thoracic ROM, craniovertebral angle (CVA), cranio-rotation angle (CRA), thoracic kyphosis angle (TKA), proprioception, and the Neck Disability Index (NDI). Paired *t*-tests were used for within-group comparisons, and independent *t*-tests were used to compare change scores between groups. The Benjamini–Hochberg false discovery rate (FDR) procedure was applied to the between-group comparisons to account for multiple testing. *Results:* The TFE group showed significant improvements in all outcome measures following the intervention (*p* < 0.001). The CSE group showed significant improvements in pain, cervical flexion, extension, and right and left rotation, CVA, CRA, proprioception during extension and right and left rotation, and NDI (*p* < 0.05); however, no significant improvements were observed in thoracic ROM, TKA, or proprioception during flexion (*p* > 0.05). After FDR correction, between-group comparisons demonstrated significantly greater improvements in the TFE group than in the CSE group across all outcome measures (q < 0.05). *Conclusions:* TFE may be more effective than CSE in improving pain, ROM, spinal alignment, proprioception, and neck-related disability in patients with neck pain and FHP.

## 1. Introduction

Forward head posture (FHP) is a sagittal-plane postural deviation in which the head is positioned anteriorly relative to a vertical reference line through the trunk [1]. FHP is commonly observed among individuals engaged in prolonged screen-based activities and has been associated with chronic neck pain and neck-related disability [2,3]. Biomechanically, FHP may involve excessive lower cervical flexion and compensatory upper cervical extension [4]. This altered alignment increases the moment arm of the head and may increase mechanical loading on cervical structures, potentially contributing to pain and restricted range of motion (ROM) [4,5]. Furthermore, because cervical muscle spindles play an important role in head and neck position sense, persistent alterations in cervical muscle length and activation patterns may modify proprioceptive input and contribute to impaired postural control [6,7,8].

Cervical stabilization exercise (CSE) is commonly used in individuals with FHP and chronic neck pain [3]. CSE is typically performed as cranio-cervical flexion training using a pressure biofeedback unit and aims to minimize excessive activation of the superficial cervical flexors while facilitating selective activation of the deep cervical flexors [9]. The deep cervical flexors contribute to segmental stability and postural control; however, individuals with chronic neck pain may exhibit impaired activation of these muscles accompanied by increased compensatory activity of the superficial cervical muscles [10,11]. Accordingly, deep cervical flexor motor-control training has been shown to improve pain and neck-related disability [12]. Nevertheless, CSE is a task-specific motor-control intervention that may require careful instruction and feedback, particularly during the early stages of training [13,14,15]. These practical demands have prompted interest in alternative or complementary interventions that are simpler to perform while providing comparable clinical benefits.

Thoracic flexibility exercise (TFE) is a form of mobility exercise that actively facilitates thoracic rotational mobility through axial rotation of the trunk and may be used as an intervention to restore restricted thoracic motion and improve cervicothoracic function [16,17]. Previous studies have applied exercises emphasizing sagittal-plane movements, such as thoracic extension, to improve thoracic mobility [18]. However, the thoracic facet joint surfaces are oriented more closely to the frontal plane than to the sagittal plane, providing morphological characteristics that are more favorable for lateral flexion and rotational movements than for sagittal-plane motion [19]. Accordingly, TFE may be considered an active mobility exercise that more directly facilitates rotational mobility in accordance with the anatomical and kinematic characteristics of the thoracic spine [16,20]. Patients with chronic neck pain exhibit reduced thoracic mobility compared with asymptomatic individuals, and decreased thoracic mobility has been reported to be associated with greater neck pain intensity [21,22]. This relationship may be explained from the perspective of regional interdependence, which proposes that dysfunction in anatomically and biomechanically related regions may influence the primary site of pain; accordingly, restricted thoracic mobility may increase mechanical demands on the cervical spine and thereby contribute to neck pain [23,24]. Cho [18] reported significant improvements in neck pain, craniovertebral angle, and neck-related disability following upper thoracic mobilization combined with mobility exercise in individuals with FHP. Seo [17] reported that thoracic mobility exercise increased cervical and thoracic range of motion and reduced neck pain and neck-related disability in office workers with chronic neck pain. Recent systematic reviews and meta-analyses have further supported the clinical relevance of thoracic spine interventions in individuals with neck pain, reporting beneficial effects of thoracic or cervicothoracic manipulation on pain and neck-related disability [25,26]. However, previous reviews have largely evaluated manipulation-based thoracic interventions, and the independent effects of active thoracic flexibility exercise remain unclear.

Despite this biomechanical rationale, the clinical effects of an independent thoracic rotation-specific exercise in individuals with chronic neck pain and FHP remain insufficiently investigated. In particular, limited evidence is available directly comparing thoracic flexibility exercise with cervical-focused motor-control intervention. Therefore, this study aimed to compare the effects of TFE and CSE on pain, neck-related disability, cervical and thoracic ROM, craniovertebral angle (CVA), cranio-rotation angle (CRA), thoracic kyphosis angle (TKA), and proprioception in individuals with chronic neck pain and FHP. We hypothesized that both interventions would improve clinical and functional outcomes, whereas TFE would produce greater improvements in thoracic rotation ROM and postural alignment than CSE.

## 2. Materials and Methods

### 2.1. Participants

To determine the appropriate sample size, an a priori power analysis was conducted using G*Power software (version 3.1.2; Franz Faul, University of Kiel, Kiel, Germany). The effect size was derived from a previous randomized clinical trial that compared a thoracic-focused intervention consisting of thoracic mobilization combined with mobility exercise with a cervical-focused intervention consisting of cervical mobilization combined with stabilization exercise in individuals with FHP [27]. Although the intervention protocol in the previous study was not identical to the TFE protocol used in the present study, it was selected because of its similarity in the target population, cervicothoracic intervention approach, and comparative study design. Based on an effect size of 1.16, a significance level of α = 0.05, and a statistical power of 0.80, the analysis indicated that a minimum of 10 participants per group was required. Considering a potential dropout rate of 20%, a total of 24 participants were recruited.

The inclusion criteria were as follows: (1) a CVA of 51° or less, (2) nonspecific neck pain persisting for at least 3 months, (3) a neck pain intensity score of 3–6 on the numeric pain rating scale (NPRS), and (4) the ability to understand the study procedures and objectives and provide voluntary informed consent. The exclusion criteria were as follows: (1) a history of trauma or surgery involving the cervical or thoracic spine, (2) whiplash-associated disorder, radiating pain in the upper extremity, cervical myelopathy, cervical radiculopathy, a positive Lhermitte’s sign, or other neurological signs suggestive of central or peripheral nervous system involvement, such as hyperreflexia, sensory deficits, hand muscle atrophy, nystagmus, visual disturbance, or pathological reflexes, (3) vascular disorders involving the head and neck region, (4) red-flag conditions, including fracture, tumor, osteoporosis, rheumatoid arthritis, or metabolic disease, (5) uncontrolled hypertension or a history of long-term corticosteroid use, and (6) psychiatric disorders or inability to understand and follow the study instructions. A recruitment notice was used to openly enlist participants among patients admitted to Kang-il Hospital. A total of 24 participants voluntarily agreed to participate in this study and met all inclusion and exclusion criteria; thus, all 24 were enrolled. This study was conducted as an assessor-blinded, two-arm, parallel-group randomized controlled trial.

After all 24 participants had been enrolled and completed the baseline assessment, random allocation was performed by the principal investigator, who was not involved in outcome assessment. A random number was generated for each participant using the RAND function in Microsoft Excel. The participants were then ranked in ascending order according to the generated random numbers; the first 12 participants were allocated to the TFE group and the remaining 12 participants were allocated to the CSE group. The principal investigator was responsible for participant enrollment and randomization, whereas outcome assessments were performed by a separate assessor. Because randomization was conducted only after enrollment and baseline assessment of all participants had been completed, group assignments were not available during the enrollment or baseline assessment process. No sealed-envelope allocation procedure was used.

All participants provided written informed consent after receiving a thorough explanation of the study procedures and potential risks, including pain, falls, and other safety-related issues. They were informed of their right to withdraw from this study at any time without penalty. This study was conducted in accordance with the Declaration of Helsinki and approved by the Institutional Review Board of Kyungnam University (approval number: 1040460-A-2026-006; approval date: 12 May 2026). This study was also retrospectively registered with the Clinical Research Information Service under the identifier KCT0011998 on 15 May 2026. This study was conducted in accordance with the CONSORT 2025 guidelines [28].

### 2.2. Experimental Procedures

The TFE group performed TFE in the kneeling position, whereas the CSE group performed cranio-cervical flexion exercises using a pressure biofeedback unit. Both groups participated in the intervention once daily, three times per week, for a total of 4 weeks. All interventions were administered by a physical therapist with more than 5 years of clinical experience. The intervention was discontinued if a participant reported discomfort or worsening pain during exercise or requested discontinuation of the intervention. To minimize potential confounding effects, participants were instructed not to perform any additional exercises other than the assigned intervention during the study period. The content and duration of the exercises were strictly aligned with a predefined protocol to ensure consistency between the two groups. Baseline characteristics, including age, height, body mass, sex, body mass index, neck pain intensity, and neck disability, were collected for all participants. Pre-intervention assessments were conducted one day before the intervention, and post-intervention assessments were performed the day after the final session. To prevent detection bias, all outcome measurements were performed by an independent assessor who was blinded to the participants’ group allocations. Neck pain intensity was assessed using the NPRS, and cervical range of motion was measured using a digital dual inclinometer and universal goniometer. Spinal alignment was assessed using photographs captured with a digital camera (Nikon D5300, Nikon Corporation, Tokyo, Japan) and analyzed using image analysis software (ImageJ 1.54, National Institutes of Health, Bethesda, MD, USA). Proprioception was assessed using the joint position error test. Neck-related disability was assessed using the Neck Disability Index (NDI). The total time required to complete all assessments was approximately 30 min per participant.

### 2.3. Thoracic Flexibility Exercise

**Figure 1:**
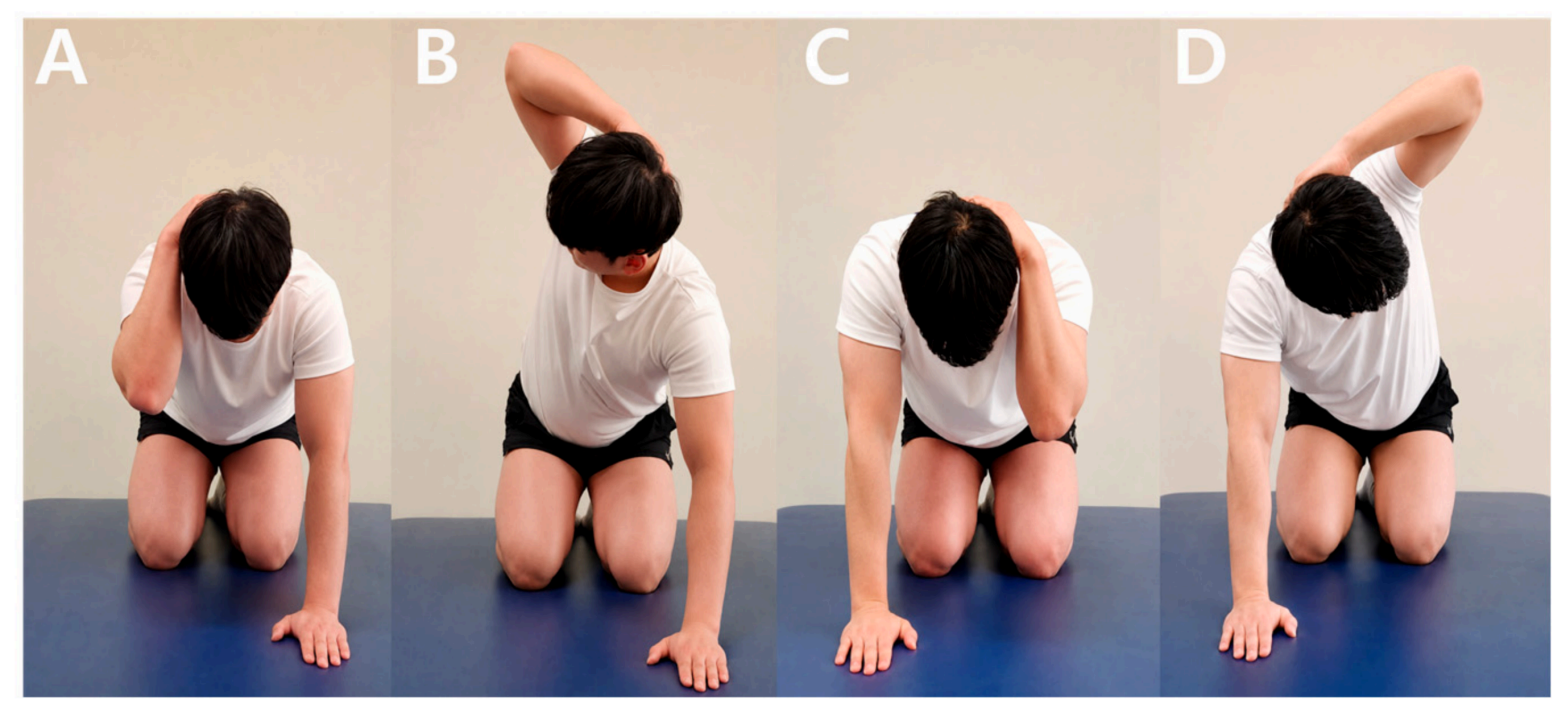
Thoracic flexibility exercise. (**A**,**C**) starting position; (**B**,**D**) ending position.

TFE was performed in the kneeling position to prevent compensatory lumbar movement during exercise, as described in a previous study [29]. The starting position was a kneeling position with the hips in contact with the heels. One hand was placed on the floor for support, while the opposite hand was positioned behind the head. From this position, participants lifted the elbow and rotated the trunk as far as possible toward the end range of thoracic rotation. The end position was maintained for 10 s, after which participants slowly returned to the starting position. To minimize compensatory movements, the therapist monitored the pelvis and lumbar spine through palpation and provided verbal and tactile feedback as needed. Each set consisted of 10 repetitions to the left and 10 repetitions to the right. Participants performed two sets, with a 5 s rest between repetitions and a 30 s rest between sets. To ensure consistent exercise timing, an interval timer application on a smartphone was used to control the duration of each exercise and rest interval (Figure 1).

### 2.4. Cervical Stabilization Exercise

**Figure 2:**
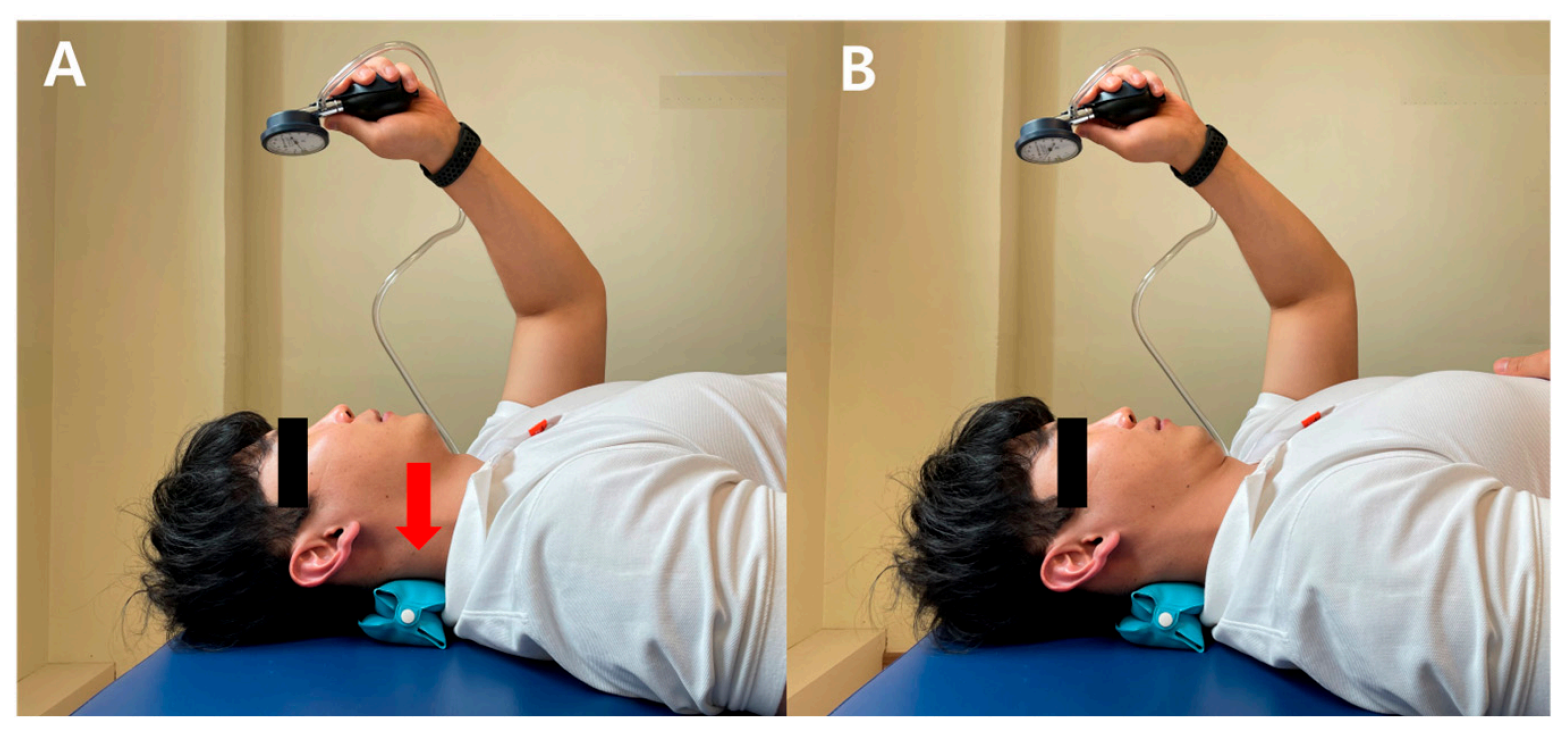
Cervical stabilization exercise. (**A**) starting position; (**B**) ending position. The arrow indicates the direction of the chin-tuck movement.

CSE was performed using a pressure biofeedback unit (Stabilizer, Chattanooga Group, Inc., Hixson, TN, USA) based on the protocol described by a previous study [30]. Participants began in the supine position, and a pressure biofeedback unit was placed under the neck and inflated to a baseline pressure of 20 mmHg. They performed a chin tuck to progressively reach five target pressure levels (22, 24, 26, 28, and 30 mmHg), holding each level for 10 s. One set comprised the five target pressure levels, and four sets were completed with 5 s of rest between repetitions and 30 s of rest between sets. The therapist provided palpation-based monitoring, along with verbal and tactile feedback, to limit compensatory movements. Exercise and rest intervals were controlled using a smartphone interval timer (Interval Timer Plus, Su-Au-Hwang, Taipei, Taiwan) (Figure 2).

### 2.5. Outcome Measurements

#### 2.5.1. Pain

Pain intensity was assessed using the NPRS. The NPRS is an 11-point scale ranging from 0 (no pain) to 10 (the worst imaginable pain), and participants were instructed to select the number that best represented their perceived level of neck pain. The NPRS has demonstrated moderate to excellent test–retest reliability, with intraclass correlation coefficient (ICC) = 0.58–0.93 [31].

#### 2.5.2. Range of Motion

Cervical and thoracic ROM were assessed using a digital dual inclinometer (Dualer IQ, JTECH Medical Industries, Inc., Midvale, UT, USA) and a goniometer (Universal goniometer, Dongmyeong, Seoul, Republic of Korea). All measurements were performed in triplicate, and the mean value was used for subsequent statistical analysis. Cervical flexion and extension ROM were assessed using a digital dual inclinometer with participants in a neutral standing posture. Following zero calibration, one sensor was positioned over the T1 spinous process, while the other was placed over the occiput. Participants were instructed to perform maximal active cervical flexion and extension. This measurement protocol using a digital dual inclinometer has demonstrated excellent intra- and inter-rater reliability, with ICC = 0.92–0.96 [32]. Cervical rotation ROM was assessed using a universal goniometer with the participant in a seated position. Following zero calibration, the goniometer axis was centered over the vertex of the head, with the stationary arm aligned parallel to the sagittal plane and the moving arm aligned with the tip of the nose. Participants were instructed to perform maximal active cervical rotation to both the right and left, ensuring that compensatory trunk or shoulder movements were avoided. The final rotation angle was recorded at the end range of each movement. This measurement protocol has demonstrated excellent intra- and inter-rater reliability, with ICC = 0.79–0.98 [33].

Thoracic flexion and extension were assessed using the same dual inclinometer configuration, with sensors positioned over the T1–T2 and T11–T12 spinous processes, respectively. Participants were instructed to perform maximal active thoracic flexion and extension while maintaining a neutral standing posture. This measurement protocol demonstrated strong to excellent reliability, with ICC ≥ 0.80 [34]. Thoracic rotation ROM was assessed with participants in a seated position, with hips and knees flexed to 90°. A 21 cm ball was placed between the knees to minimize pelvic rotation and ensure isolated thoracic movement. Participants crossed their arms over the chest and performed maximal active trunk rotation to each side. During the movement, a universal goniometer was positioned parallel to the ground at the T1–T2 spinous process level, using the spine of the scapula as the primary anatomical reference point. The stationary arm was directed away from the direction of rotation, maintaining alignment with the starting position, while the moving arm tracked the rotation by remaining parallel to the spine of the scapula. The final thoracic rotation angle was recorded at the end range of each movement. This protocol has demonstrated high intra- and inter-rater reliability, with ICC = 0.85–0.95 [35].

#### 2.5.3. Spinal Alignments

Cervical alignment was assessed using a digital camera and Image J software. Participants stood in a relaxed posture at a distance of 1.5 m from the camera with the dominant side facing the lens. Camera height was adjusted so that the lens center aligned with the acromion. CVA was defined as the angle formed between a horizontal line passing through C7 and a line connecting C7 to the tragus. CRA was defined as the angle formed between the line connecting C7 to the tragus and the line connecting the tragus to the lateral canthus. This measurement protocol has demonstrated excellent reliability (ICC = 0.89–0.94) [36].

TKA was assessed using a smartphone-based inclinometer application (Goniometer Pro for Android, Digiflex Labs, Seattle, WA, USA). Participants were instructed to maintain an upright standing posture with knees extended. To minimize upper extremity interference, the shoulders and elbows were positioned in 90° of flexion. The T1 and T12 spinous processes were identified by palpation and marked. Following calibration of the smartphone inclinometer to 0° at the T1 level, the device was placed over the T12 spinous process, and the recorded angle was documented as the TKA. This smartphone-based method has demonstrated high validity compared with the radiographic Cobb method (ICC = 0.89) and shows excellent intra- and inter-rater reliability (ICC = 0.88–0.91) [37].

#### 2.5.4. Proprioception

Cervical proprioception was assessed using the joint position error test. Participants were seated 90 cm from a wall-mounted target, wearing a laser-mounted headband aligned between the eyes. After aligning the laser with the target center, participants performed active cervical movements—flexion, extension, and rotation—with their eyes closed, returning to their perceived neutral head position. Upon verbally signaling the return to the starting position, the examiner marked the final laser point on the target. The joint position error test was defined as the distance between the target center and the recorded laser point. This protocol has demonstrated good to excellent test–retest reliability (ICC = 0.77–0.87) [38].

#### 2.5.5. Neck Disability Index

The NDI was used to assess self-reported functional disability associated with neck pain. It consists of 10 items: pain intensity, personal care, lifting, reading, headaches, concentration, work, driving, sleeping, and recreation. Each item is rated on a 6-point Likert scale ranging from 0 to 5, yielding a total score ranging from 0 to 50. The NDI has demonstrated moderate to excellent test–retest reliability (ICC = 0.64–0.92) [39].

### 2.6. Statistical Analysis

Statistical analysis was performed using SPSS version 25.0 (SPSS Inc., Chicago, IL, USA). The normality of the data distribution was assessed using the Shapiro–Wilk test. Baseline characteristics were compared between groups using independent *t*-tests for continuous variables and chi-square tests for categorical variables. All measured variables were normally distributed (*p* > 0.05). To analyze the effects of the interventions, paired *t*-tests were used to examine within-group changes from pre- to post-intervention, and independent *t*-tests were used to compare the mean change scores between the TFE and CSE groups. To account for multiple comparisons across the 17 outcome variables, the Benjamini–Hochberg false discovery rate (FDR) procedure was applied to the *p*-values obtained from the between-group comparisons. Statistical significance for between-group comparisons was determined based on FDR-adjusted *p*-values (q < 0.05), whereas statistical significance for within-group comparisons was set at *p* < 0.05. Within-group effect sizes were calculated using Cohen’s dz, defined as the mean pre–post change divided by the standard deviation of the paired change scores. Effect sizes were interpreted according to Cohen’s conventions, with values of 0.20, 0.50, and 0.80 representing small, moderate, and large effects, respectively [40].

## 3. Results

**Figure 3:**
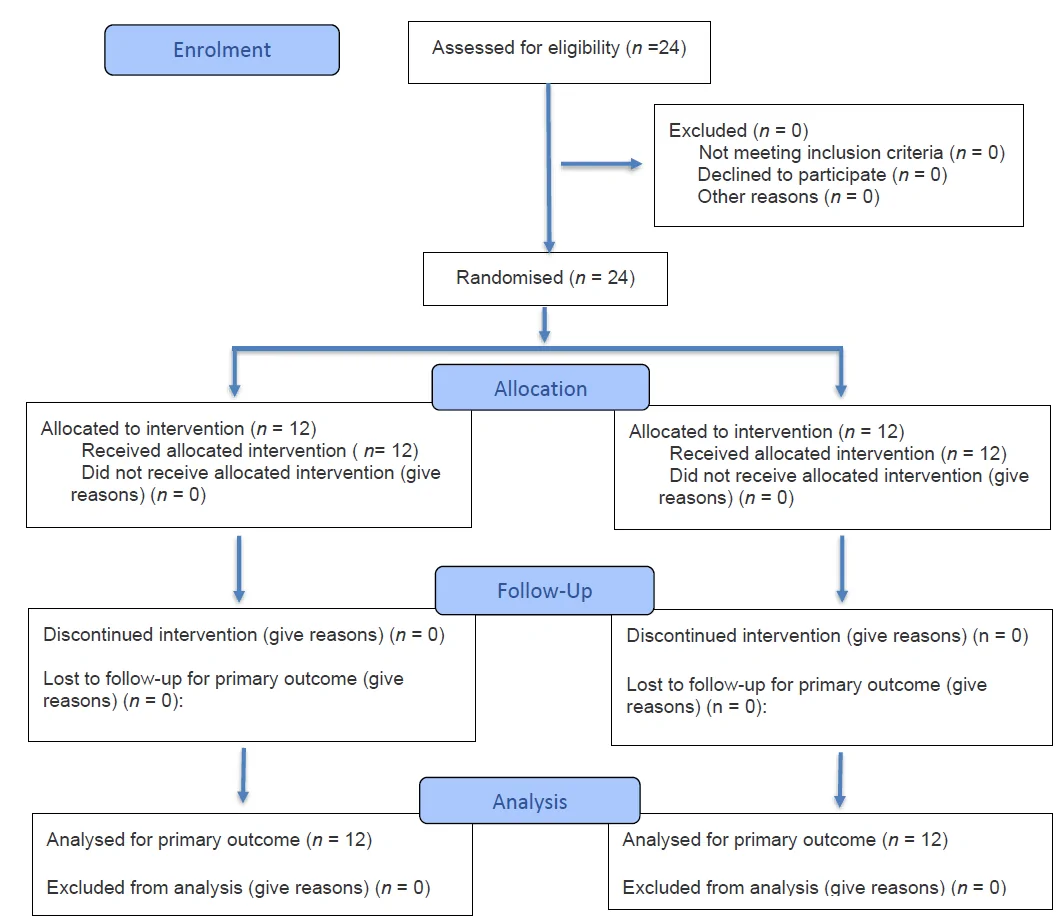
CONSORT Flow diagram.

A total of 24 participants were enrolled and randomly assigned to either the TFE group (*n* = 12) or the CSE group (*n* = 12). All participants received the allocated intervention and completed the four-week study, with no losses to follow-up or exclusions after randomization. All 24 participants were included in the final analysis. Participant flow throughout the study is presented in Figure 3. The general and clinical characteristics of the participants at baseline are summarized in Table 1. No significant between-group differences were observed in baseline characteristics (*p* > 0.05).

Table 2 presents the measurement values obtained for all outcome variables. Within-group comparisons using a paired *t*-test revealed that the TFE group exhibited significant improvements across all measures of pain, ROM, spinal alignment, proprioception, and the NDI (*p* < 0.001). In contrast, the CSE group showed no significant changes in thoracic ROM, TKA, and proprioception for flexion (*p* > 0.05), although significant improvements were observed in the remaining variables (*p* < 0.05). Furthermore, after applying the Benjamini–Hochberg false discovery rate (FDR) correction to the 17 between-group comparisons, the TFE group demonstrated significantly greater improvements than the CSE group across all outcome variables (*q* < 0.05). These findings indicate that the magnitude of improvement differed significantly between the two intervention groups across the measured outcomes.

## 4. Discussion

This study investigated the effects of TFE on pain, function, and spinal alignment in patients with neck pain and FHP. Specifically, the effects of TFE on neck pain, cervical and thoracic ROM, spinal alignment, proprioception, and NDI were evaluated. Direct comparisons between TFE and CSE remain limited. Significant between-group differences favoring TFE were observed across all measured outcomes after FDR correction (q < 0.05). These findings support the hypothesis that TFE is more effective than CSE in producing short-term improvements in most measured outcomes related to neck pain, physical function, and spinal alignment.

In this study, NPRS scores decreased by 3.75 points in the TFE group and by 2.50 points in the CSE group, indicating a greater reduction in pain intensity following TFE. One possible explanation for this finding is that the increase in thoracic mobility following TFE reduced the need for compensatory cervical motion. In addition, the reduction in thoracic kyphosis may have mitigated excessive lower cervical flexion, thereby reducing mechanical demands on the neck and shoulder regions and contributing to pain relief. Joshi et al. suggested that the cervical and thoracic regions are interdependent and that increased thoracic kyphosis restricts thoracic mobility, thereby increasing the demand for compensatory cervical motion. Altered spinal alignment may also change the length–tension relationships of the surrounding muscles and soft tissues, thereby increasing mechanical loading and contributing to pain [21]. Cho [18] reported a reduction in neck pain following thoracic mobility exercises in patients with neck pain and FHP, and Cleland [41] demonstrated that thoracic mobilization was effective in reducing neck pain. Although these previous studies used thoracic mobility exercises or mobilization rather than the specific TFE protocol applied in the present study, their findings similarly suggest that interventions targeting the thoracic spine may improve thoracic mobility and alignment, reduce mechanical demands on the cervical region, and contribute to pain relief. Importantly, the 3.75-point reduction in NPRS observed in the TFE group exceeded the reported MCID of 1.5 points, indicating that the observed improvement may be clinically meaningful in addition to being statistically significant [31].

Regarding cervical ROM, flexion increased by 11.84° in the TFE group and by 6.70° in the CSE group, while extension increased by 15.50° and 7.50°, respectively. Right cervical rotation increased by 16.10° in the TFE group and by 7.90° in the CSE group, whereas left cervical rotation increased by 16.70° and 6.30°, respectively. These findings indicate consistently larger improvements in cervical ROM following TFE. Similarly, thoracic flexion increased by 12.70° in the TFE group and by 3.40° in the CSE group, while thoracic extension increased by 11.60° and 1.90°, respectively. Right thoracic rotation increased by 16.10° in the TFE group and by 3.30° in the CSE group, whereas left thoracic rotation increased by 15.90° and 2.10°, respectively. These results demonstrate greater improvements in thoracic mobility following TFE, particularly in rotational ROM. Because the cervical and thoracic regions function as an integrated biomechanical chain, reduced thoracic mobility may increase the need for compensatory cervical motion and increase tension in the surrounding soft tissues [42]. Therefore, improvements in thoracic mobility may have reduced compensatory mechanical demands on the cervical spine and facilitated greater cervical ROM. Furthermore, CSE in the present study primarily emphasized sagittal-plane control, whereas TFE incorporated rotational and multiplanar movements. TFE may therefore have promoted multiplanar spinal mobility and soft-tissue flexibility, thereby facilitating movement throughout the cervicothoracic region [29]. Seo [17] reported significant increases in thoracic ROM following thoracic mobility exercises in patients with neck pain, and Joshi [43] demonstrated improvements in cervical ROM after applying thoracic mobilization and mobility exercises. These findings are consistent with the results of the present study.

In terms of spinal alignment, CVA increased by 7.22° in the TFE group and by 3.90° in the CSE group. CRA decreased by 6.92° in the TFE group and by 4.29° in the CSE group, whereas TKA decreased by 7.10° and 1.70°, respectively. These findings indicate greater improvements in CVA, CRA, and TKA following TFE. FHP is characterized by an anterior position of the head relative to the body’s line of gravity, resulting in increased upper cervical extension and lower cervical flexion. This postural pattern is frequently accompanied by increased upper thoracic kyphosis [44,45]. Thoracic rotation is accompanied by coupled segmental movements, including lateral flexion and facet-joint gliding, which may facilitate thoracic mobility [42]. Accordingly, the rotational movements incorporated into TFE may have facilitated thoracic mobility and contributed to the observed reduction in thoracic kyphosis. This reduction may, in turn, have decreased the postural demands for excessive lower cervical flexion and upper cervical extension, thereby contributing to the simultaneous improvements in both CVA and CRA. Ferraz [46] suggested that improving cervical and thoracic alignment may enhance postural control and reduce compensatory load on the cervical region. Furthermore, Cho [18] reported a significant increase in CVA together with a reduction in upper thoracic kyphosis after thoracic mobility exercises were applied to patients with FHP. These findings suggest that interventions designed to enhance thoracic mobility may promote favorable alignment changes throughout the cervicothoracic region, consistent with the results of the present study. Although an established MCID for CVA is currently unavailable, the 7.22° increase observed in the TFE group exceeded previously reported MDC values of approximately 2.6–2.8°, suggesting that the observed postural change was greater than measurement error [36].

Comparison of the pre- and post-intervention changes showed that joint position error during flexion decreased by 5.13 cm in the TFE group and by 1.79 cm in the CSE group. During extension, the corresponding reductions were 6.06 cm and 3.30 cm, respectively. Joint position error during right rotation decreased by 6.43 cm in the TFE group and by 3.20 cm in the CSE group, while that during left rotation decreased by 7.52 cm and 4.22 cm, respectively. Overall, the magnitude of reduction in joint position error was greater in the TFE group. FHP shifts the head’s center of mass anterior to the line of gravity, thereby increasing the flexion moment in the cervical spine and inducing a compensatory increase in the tension of the upper trapezius and posterior cervical muscles to counteract this effect [47]. Consequently, the central nervous system inaccurately estimates the current position of the head, resulting in an increased joint position error [48]. The CVA serves as a quantitative index of forward head posture severity, where a smaller angle denotes a more anteriorly positioned head [49]. In the present study, the TFE group demonstrated a significant increase in the CVA compared to the CSE group. This suggests that realigning the head’s center of mass closer to the line of gravity reduced compensatory hypertonicity in the posterior cervical muscles and optimized the length–tension relationships of the muscles surrounding the cervical spine, thereby improving the accuracy of proprioceptive afferent input. A prior study by Yong [50] reported that an increase in the CVA is accompanied by a reduction in cervical joint position error, demonstrating a significant negative correlation between these two variables. Furthermore, Abdel-Aziem [51] demonstrated that individuals with FHP exhibit a markedly increased joint position error across all directions of cervical motion compared to healthy controls, owing to the persistent hypertonicity of the posterior cervical muscles. These findings align with the biomechanical mechanisms identified in the present study and strongly support the validity of our results, confirming that the restoration of spinal alignment via TFE optimizes the mechanical environment of the muscles surrounding the cervical spine, ultimately enhancing proprioceptive control.

For NDI, scores decreased by 11.41 points in the TFE group and by 7.50 points in the CSE group, indicating a larger reduction in neck-related disability following TFE. The NDI assesses self-reported neck-related disability across several domains, including pain intensity, personal care, reading, headaches, concentration, work, driving, sleep, and recreation [52]. Activities involving visual scanning or upper extremity use often require coordinated movements of the eyes, head, cervical spine, and trunk rather than isolated cervical motion [42]. Accordingly, reductions in pain, broader improvements in cervical and thoracic ROM, and favorable changes in spinal alignment may have collectively contributed to the greater reduction in NDI. The greater improvement in neck disability observed in the TFE group may therefore reflect the combined effects of greater increases in ROM, enhanced proprioception, reduced pain, and improved spinal alignment compared with the CSE group. Puntumetakul [53] reported a significant reduction in NDI following thoracic manipulation in patients with mechanical neck pain. Similarly, McDevitt [54] suggested that interventions targeting the thoracic spine may reduce neck-related disability. Although the intervention methods used in these studies differed from the TFE protocol applied in the present study, their findings support the view that improving thoracic mobility may reduce mechanical demands on the cervical region and contribute to improved neck-related function. Importantly, the 11.41-point reduction in NDI observed in the TFE group exceeded the reported MCID of 5.5 points as well as previously reported MDC values of approximately 6.9–10.5 points, suggesting that the reduction in neck-related disability was clinically meaningful and exceeded measurement error [55]. Taken together, these findings suggest that TFE produced clinically relevant short-term improvements in pain and neck-related disability, together with a postural change exceeding reported measurement-error thresholds.

This study has several limitations. First, the participants were limited to patients with neck pain and FHP. Therefore, caution should be exercised when generalizing the findings to individuals with nonspecific neck pain without FHP or to patients with neck pain associated with specific pathological conditions. Second, the intervention period was relatively short at four weeks, and the long-term effects of TFE could not be determined because no follow-up assessments were conducted. Third, although the sample size met the minimum requirement determined by the a priori power analysis using G*Power, the effect size used for the calculation was derived from a previous study involving a related but not identical intervention. Therefore, the required sample size may have been underestimated. In addition, the relatively small sample size may have limited the precision of the effect estimates and the generalizability of the findings. Furthermore, the large within-group Cohen’s dz values should be interpreted cautiously because they represent standardized pre–post changes within each group and, given the small sample size, may have limited precision; they should not be interpreted as direct evidence of between-group superiority or clinical importance. Fourth, because of the nature of the exercise interventions, neither the participants nor the treating therapist could be blinded to group allocation, although the outcome assessor was blinded. Therefore, potential performance- or expectation-related bias cannot be completely excluded. Finally, because the participants were adults in their 20s to 40s, the findings may not be applicable to younger or older populations. Therefore, future studies should include patients with more diverse demographic and clinical characteristics, use longer intervention and follow-up periods, and recruit larger sample sizes to further confirm the effects of TFE.

## 5. Conclusions

In this randomized controlled trial, we investigated the effects of TFE on pain, cervical and thoracic ROM, spinal alignment, proprioception, and NDI in patients with neck pain and FHP. The TFE group demonstrated significantly greater improvements than the CSE group in pain intensity, cervical and thoracic ROM, CVA, CRA, TKA, proprioception, and NDI. These findings indicate that TFE, by providing multiplanar thoracic mobility and improving cervicothoracic alignment, may serve as a clinically valuable alternative to conventional CSE in enhancing functional mobility and sensorimotor control during neck pain rehabilitation.

## Figures and Tables

**Table 1:** General and clinical characteristics of participants at baseline (*N* = 24).

Characteristics	TFE Group (*n* = 12)	CSE Group (*n* = 12)	χ^2^/*p*
Age (year)	36.2 ± 3.4	36.8 ± 3.4	0.676 ^b^
Height (cm)	165.5 ± 7.2	168.5 ± 7.5	0.330 ^b^
Body mass (kg)	62.8 ± 14.2	69.8 ± 12.5	0.207 ^b^
Gender Male/female (%)	7/5 (58.3/41.7)	8/4 (66.7/33.3)	0.673 ^a^
BMI (kg/m^2^)	22.7 ± 3.4	24.4 ± 2.9	0.189 ^b^
NPRS (score)	5.4 ± 0.8	5.3 ± 0.8	0.797 ^b^
CVA (°)	41.5 ± 3.2	41.2 ± 3.5	0.829 ^b^
NDI (score)	18.3 ± 3.2	18.2 ± 4.3	0.917 ^b^

Abbreviations: TFE = thoracic flexibility exercise, CSE = cervical stabilization exercise, BMI = body mass index, NPRS = numeric pain rating scale, CVA = craniovertebral angle, NDI = neck disability index. Values are expressed as mean ± standard deviation or n (%): ^a^ chi-square test, ^b^ independent *t*-test.

**Table 2:** Results of statistical analysis for variables (*N* = 24).

Parameters	TFE Group (*n* = 12)					CSE Group (*n* = 12)					FDR q
	Pre	Post	Change Score (CI)	*t (p)*	ES	Pre	Post	Change Score (CI)	*t (p)*	ES	
NPRS (score)	5.41 ± 0.79	1.67 ± 0.65	−3.75 (−4.30, −3.19)	−15.00 * (<0.001)	4.33	5.33 ± 0.78	2.83 ± 1.53	−2.50 (−3.42, −1.58)	−5.98 * (<0.001)	1.73	0.022 ^†^
Cervical ROM											
Flexion (°)	42.10 ± 6.50	53.94 ± 5.40	11.84 (9.18, 14.51)	9.78 * (<0.001)	2.82	41.50 ± 7.10	48.20 ± 6.20	6.70 (2.66, 10.74)	3.65 * (0.004)	1.05	0.038 ^†^
Extension (°)	48.60 ± 8.19	64.10 ± 7.01	15.50 (10.87, 20.13)	7.36 * (<0.001)	2.13	47.90 ± 7.50	55.40 ± 6.80	7.50 (3.17, 11.83)	3.81 * (0.003)	1.10	0.024 ^†^
Rt. Rotation (°)	56.40 ± 8.50	72.50 ± 6.19	16.10 (11.49, 20.71)	7.68 * (<0.001)	2.22	55.80 ± 9.00	63.70 ± 7.50	7.90 (2.86, 12.94)	3.45 * (0.005)	1.00	0.025 ^†^
Lt. Rotation (°)	55.10 ± 9.10	71.80 ± 6.80	16.70 (11.74, 21.66)	7.41 * (<0.001)	2.14	56.20 ± 8.20	62.50 ± 8.00	6.30 (1.42, 11.18)	2.84 * (0.016)	0.82	0.010 ^†^
Thoracic ROM											
Flexion (°)	28.50 ± 5.40	41.20 ± 5.49	12.70 (9.42, 15.99)	8.51 * (<0.001)	2.46	29.10 ± 6.00	32.50 ± 5.80	3.40 (−0.16, 6.96)	2.10 (0.059)	0.61	<0.001 ^†^
Extension (°)	18.20 ± 4.49	29.80 ± 4.20	11.60 (8.97, 14.23)	9.72 * (<0.001)	2.81	19.50 ± 4.80	21.40 ± 5.10	1.90 (−1.09, 4.89)	1.39 (0.189)	0.40	<0.001 ^†^
Rt. Rotation (°)	26.40 ± 5.80	42.50 ± 5.10	16.10 (12.79, 19.41)	10.71 * (<0.001)	3.09	25.80 ± 6.20	29.10 ± 6.50	3.30 (−0.53, 7.13)	1.89 (0.084)	0.55	<0.001 ^†^
Lt. Rotation (°)	25.90 ± 6.10	41.80 ± 5.40	15.90 (12.41, 19.39)	10.03 * (<0.001)	2.90	26.50 ± 5.50	28.60 ± 5.90	2.10 (−1.34, 5.54)	1.34 (0.206)	0.39	<0.001 ^†^
Spinal alignment											
CVA (°)	41.50 ± 3.19	48.71 ± 3.20	7.22 (5.08, 9.35)	7.45 * (<0.001)	2.15	41.20 ± 3.50	45.10 ± 3.20	3.90 (1.87, 5.93)	4.24 * (0.001)	1.22	0.022 ^†^
CRA (°)	156.08 ± 4.63	149.14 ± 6.23	−6.92 (−8.93, −4.94)	−7.66 * (<0.001)	2.21	156.45 ± 4.43	152.16 ± 4.43	−4.29 (−6.33, −2.25)	−4.86 * (0.001)	1.40	0.038 ^†^
TKA (°)	49.20 ± 4.49	42.10 ± 4.00	−7.10 (−9.67, −4.52)	−6.07 * (<0.001)	1.75	48.50 ± 4.80	46.80 ± 4.50	−1.70 (−4.51, 1.11)	−1.33 (0.209)	0.38	0.012 ^†^
Proprioception											
Flexion(cm)	7.41 ± 2.89	2.28 ± 0.60	−5.13 (−7.00, −3.26)	−6.04 * (<0.001)	1.74	6.93 ± 3.25	5.14 ± 2.53	−1.79 (−3.71, 0.12)	−2.06 (0.064)	0.59	0.022 ^†^
Extension (cm)	8.87 ± 4.02	2.81 ± 1.00	−6.06 (−8.54, −3.57)	−5.37 * (<0.001)	1.55	8.62 ± 2.91	5.32 ± 2.20	−3.30 (−4.42, −2.18)	−6.51 * (<0.001)	1.88	0.038 ^†^
Rt. Rotation (cm)	9.62 ± 4.99	3.19 ± 1.37	−6.43 (−9.16, −3.69)	−5.16 * (<0.001)	1.49	9.28 ± 2.12	6.08 ± 1.55	−3.20 (−4.85, −1.55)	−4.27 * (0.001)	1.23	0.038 ^†^
Lt. Rotation (cm)	10.00 ± 2.83	2.48 ± 0.60	−7.52 (−9.13, −5.91)	−10.28 * (<0.001)	2.97	10.29 ± 4.51	6.08 ± 2.94	−4.22 (−7.09, −1.34)	−3.23 * (0.008)	0.93	0.037 ^†^
NDI (score)	18.33 ± 3.20	6.92 ± 3.12	−11.41 (−14.70, −8.14)	−7.66 * (<0.001)	2.21	18.17 ± 4.43	10.67 ± 4.70	−7.50 (−9.83, −5.16)	−7.06 (<0.001)	2.04	0.024 ^†^

Abbreviations: TFE = thoracic flexibility exercise, CSE = cervical stabilization exercise, change score = post-test – pre-test, CI = 95% confidence interval, ES = within-group effect size (Cohen’s dz), FDR = false discovery rate, q = FDR-adjusted *p*-value, NPRS = numeric pain rating scale, ROM = range of motion, Rt. = right, Lt. = left, CVA = craniovertebral angle, CRA = cranio-rotation angle, TKA = thoracic kyphosis angle, NDI = neck disability index. Values are expressed as mean ± standard deviation. * Significant difference between pre- and post-intervention within the group by the paired *t*-test. ^†^ Significant difference between groups based on the Benjamini–Hochberg FDR-adjusted *p*-value (q < 0.05).

## Data Availability

The original data presented in the study are openly available in FigShare at https://doi.org/10.6084/m9.figshare.33033515.

## Abbreviations

The following abbreviations are used in this manuscript:

FHP	Forward head posture
TFE	Thoracic flexibility exercise
CSE	Cervical stabilization exercise
NPRS	Numeric pain rating scale
FDR	Benjamini–Hochberg false discovery rate
ROM	Range of motion
CVA	Craniovertebral angle
CRA	Cranio-rotation angle
TKA	Thoracic kyphosis angle
NDI	Neck Disability Index
ICC	Intraclass correlation coefficient
ES	Effect size
